# Enhancing the Ethical Conduct of HIV Research with Migrant Sex Workers: Human Rights, Policy, and Social Contextual Influences

**DOI:** 10.1371/journal.pone.0155048

**Published:** 2016-05-09

**Authors:** Shira M. Goldenberg, Kimberly C. Brouwer, Teresita Rocha Jimenez, Sonia Morales Miranda, Monica Rivera Mindt

**Affiliations:** 1 Faculty of Health Sciences, Simon Fraser University, 8888 University Drive, Burnaby, BC, V5A 1S6, Canada; 2 Gender and Sexual Health Initiative, British Columbia Centre for Excellence in HIV/AIDS, Department of Medicine, University of British Columbia, 608–1081 Burrard Street (St. Paul's Hospital), Vancouver, BC, V6Z 1Y6, Canada; 3 HIV Prevention Research Ethics Institute, Fordham University, 441 E. Fordham Road, Bronx, NY, 10458, United States of America; 4 Division of Global Public Health, University of California San Diego, 9500 Gilman Drive, La Jolla, CA, 92093–0507, United States of America; 5 HIV Unit, Universidad del Valle, Guatemala City, Guatemala; 6 Department of Psychology, Fordham University, 441 E. Fordham Road, Bronx, NY, 10458, United States of America; University of Athens, Medical School, GREECE

## Abstract

**Background:**

Migrant sex workers are often highly marginalized and disproportionately experience health and social inequities, including high prevalence of HIV, sexually transmitted infections, and human rights violations. In recent years, research involving migrant sex workers has increased, yet many knowledge gaps remain regarding how best to protect research participant rights and welfare. Our objective was to identify key challenges and opportunities related to the responsible conduct of HIV research with migrant sex workers.

**Methods:**

Focus groups and interviews conducted with 33 female sex workers ≥18 years old at the Guatemala-Mexico border from June 2013–February 2014 were analyzed. Participants were recruited through community outreach by a local HIV prevention organization to sex work establishments such as bars, hotels, street corners, and truck stops.

**Results:**

Key themes influencing research engagement for migrant sex workers included researcher mistrust and fear related to research participation, rooted in the social isolation frequently faced by recent migrants; intersecting concerns related to immigration status, fear of criminalization, and compliance with sex work regulations; and perceived benefits and risks of HIV/STI testing for migrants (e.g., immigration implications, stigma) represent potential barriers and opportunities for the responsible conduct of research involving migrant sex workers.

**Conclusions:**

Results highlight the intersection between the human rights vulnerabilities of migrant sex workers and barriers to research participation, including social isolation of migrants and policy/legal barriers related to immigration and sex work. Findings illustrate the need for researchers to develop population-tailored procedures to address fears related to immigration and criminalization, and to reinforce positive and non-stigmatizing relationships with migrant sex workers. Community-led efforts to reduce stigma and foster community organization and supports for migrant sex workers are recommended, as are broader policy shifts that move away from punitive legal approaches towards approaches that safeguard and prioritize the human rights of migrant sex workers.

## Introduction

Migrant populations–that is, individuals who move across international and national borders for diverse reasons (e.g., economic, forced)–often face significant inequities related to HIV, sexually transmitted infections (STIs), and other health and human rights concerns, particularly in low- and middle-income countries (LMIC).[1–8] Migrant workers often fill low-paying positions in the informal sector, where they frequently face insecurity and unsafe working conditions, including lower access to occupational health and safety.[9–11]

Migrant women are often overrepresented is the sex industry, where they often face enhanced marginalization and health and social inequities, including HIV and STIs, violence, and human rights violations.[12–20] Sex workers’ health is shaped by structural factors including work environments, criminalization, collectivization and community participation, stigma, economic factors, and housing[21–28]; violence, as a product of gendered, economic and other structural inequalities, also shapes other health risks for sex workers, including HIV, mental health, and reduced access to care[21, 29–31]. Previous research indicates that in many settings, migrant women who engage in sex work face unique health-related harms and structural vulnerabilities, including poor healthcare access, poverty and debt, social isolation and exclusion, unsafe working conditions, stigma and discrimination related to sex work and immigration status, insecure legal status, and language barriers[1, 12, 13, 17, 32–34]. Although epidemiological research with migrant sex workers has recently increased in some settings,[12, 13, 15, 16, 18, 20, 33, 35–37] ethical issues related to the conduct of health research involving migrant sex workers in diverse contexts remain poorly understood.

In light of the enhanced vulnerabilities of migrants in the sex industry, there remains a critical need for research to develop targeted, evidence-based health interventions.[12, 38] However, migrants and ethnic minority populations are often hard to reach and remain under-represented in research.[39–43] Ethical concerns related to the responsible conduct of HIV research with key populations, such as sex workers, may be exacerbated for migrants, stemming from fears related to immigration status (e.g., lack of work authorization, deportation), as well as the frequent exposure to discrimination, violence, stigma, and social isolation migrants often report.[38–41] Previously identified ethical issues in global health research with marginalized and highly vulnerable populations, such as sex workers and people who use drugs, include: concerns regarding disclosure of illicit and often stigmatizing behaviors; the potential for exploitation and lack of respect for participants by research teams; inadequate community engagement; stress and burnout among front-line research staff; and disappointment when the research fails to improve policies or working conditions.[39, 44–59] Applicability of research to the eventual implementation of interventions and programmes serving this population may be of particular concern for marginalized populations recruited for epidemiological studies. There is a paucity of work on these and other ethical concerns in HIV research with migrant women, including the unique challenges for front-line research staff working with highly vulnerable and transient populations[39, 53, 58] and the potential for research to unintentionally fuel (mis)perceptions of migrant sex workers as “vectors of disease” or as sex trafficking victims[1, 38, 60, 61]. Sex work, migrant labour, and sex trafficking have often been conflated on an ideological and political basis, and this has historically fuelled repressive policies that have undermined efforts to advance the health and human rights of sex workers.[62–64] Unfortunately, the criminalized approaches to sex work legislation and the ‘raid and rescue’ enforcement-based models resulting from this approach have done little to further the rights of migrant sex workers; rather, criminalized approaches may rarely identify trafficked individuals or result in positive outcomes for them, while contributing to mistrust, adversarial police interactions, and enhanced stigma, marginalization, and poor access to occupational health.[21, 62, 63, 65, 66] While some studies have investigated ethical issues related to the conduct of research with marginalized populations in high-income countries,[39, 53, 54, 56, 67–70] less research attention has focused on migrant sex workers, particularly in low and middle-income countries (LMIC).

Ethical issues related to the responsible conduct of global health research with marginalized populations can arise when research procedures, topics studied, and data collection tools are designed without adequate community engagement or understanding of contextual features (e.g., collective histories of trauma; local cultural practices and norms; previous interactions with researchers). To promote ethical, valid, and relevant research with marginalized groups and especially when working in new or unfamiliar contexts, there remains a need for ethical practices that are informed by participant and community needs, priorities, and perspectives, alongside deep knowledge of local social, cultural, and political conditions (e.g., organization of the sex industry, sex work and immigration policies)[44, 54, 69, 71, 72] The aim of this study was to gather empirical evidence to enhance the ethical conduct of HIV research involving migrant sex workers through qualitative interviews and group discussions investigating key challenges and opportunities from the perspectives of sex workers at the Guatemala-Mexico border.

This study was conducted in the Guatemalan border community of Tecún Umán, which is situated along the main illegal entryway from Central America into Mexico. Intense mobility characterizes this porous border, which hosts a thriving sex industry that attracts migrant clients (e.g., agricultural workers, truck drivers). Most sex workers in the area are international migrants from other Central American countries or internal Guatemalan migrants, and many international migrants in this context engage in circular mobility to Central American home countries.[73–77] Sex work is practiced locally in entertainment venues (i.e., bars and cantinas), as well as on street corners, parks, plazas, hotels, and truck stops, river area (natural border with Mexico). In Guatemala, public health regulations require sex workers in certain venues (e.g., bars, nightclubs) to register and receive regular HIV/STI testing at municipal clinics. However, a large proportion of sex workers work without registration, particularly in non-bar settings.[78] Sex workers in this region are also exposed to immense risk of violence, and Guatemala has one of the highest murder rates in the world, particularly along the northern border with Mexico.[79] Despite the confluence of risks faced by sex workers in this context, limited data exist regarding sex workers’ health in this border region, and there remains a critical need for epidemiological and intervention research to better understand and address HIV/STI risks among sex workers. Complementing prior epidemiological research with other key populations in Central America,[80–82] in recent years, our team initiated a new program of epidemiological and qualitative research on migration, sex work, HIV/STIs, and violence along the Guatemala-Mexico border[76–78] Given the lack of published reports on ethical issues associated with the conduct of this type of research in Mexico and Central America, in 2013–2014, we carried out an exploratory study to begin to develop an evidence base to inform the responsible conduct of research involving sex workers in this setting and to contribute towards the development of community guidelines for research.[46] As previously described [46], this study broadly investigated the perceived benefits and risks of participation in HIV research for sex workers at the Guatemala-Mexico border, and an earlier report from this qualitative research highlighted the importance of access to HIV/STI prevention and testing and the positive and trusting relationships between sex workers and research teams in facilitating research participation, as well as the mixed effects of control exerted by managers.[46] However, as the majority of sex workers in this context are migrants, and this previous work did not cover migration-related experiences, the current report reports upon migration-related experiences (e.g., human rights concerns, legal immigration status) and the social contexts of migrant sex workers in shaping the responsible conduct of HIV prevention research, from the perspectives of both migrant and non-migrant sex workers.

## Methods

From June 2013–February 2014, we conducted focus groups and in-depth interviews exploring ethical issues related to HIV research participation with 33 female sex workers in Tecún Umán, Guatemala, as previously described.[46] Eligibility criteria included female, ≥18 years old, reporting exchanging sex for money, drugs, shelter or goods in the past 30 days, and speaking Spanish, and able to provide informed consent. As migration-related issues frequently emerged within broader discussions of research-related barriers and facilitators involving both migrant and non-migrant sex workers alike, the analysis included both groups of participants.

The study was approved by Institutional Review Boards at the University of California, San Diego and the Ministry of Public Health and Social Assistance in Guatemala and written informed consent was obtained prior to participation. As previously described,[46] our enhanced informed consent procedures were carefully designed to maximize participant understanding of procedures and to ensure voluntary participation. Fluent Spanish speaking, female staff guided participants through the informed consent process (i.e., by highlighting key points and/or reading the form), explaining the purpose, procedures, and benefits and risks of the study. The principal investigator, and/or project coordinator were onsite to answer any questions or concerns raised by potential participants. A Community Advisory Board (CAB) of local organizations representing sex workers, HIV prevention organizations, the municipal health clinic, and women’s organizations was established to guide the study. The CAB met in advance of and following data collection to provide detailed input on research procedures, data collection instruments, interpretation of findings, and dissemination strategies.

### Data Collection Procedures

As previously described,[46] potential participants were recruited from sex work establishments such as bars, hotels, street corners, and truck stops in Tecun Uman. Guided by principles of purposive sampling,[83] we sought to recruit women who represented a range of research participation experiences (e.g., research-exposed vs. naïve), work venues (e.g., indoor vs. outdoor), migration status (e.g., international migrants, internal migrants, local residents) and age. Recruitment was conducted by trained female outreach workers from a local community-based HIV prevention organization (EDUCAVIDA) with extensive experience working with sex workers and other key populations. During outreach, outreach workers unobtrusively approached women to explain the purpose of the study and assess eligibility and interest in participating[46].

Following informed consent, women completed a brief survey eliciting information on socio-demographics (e.g., age, education), migration history (e.g., migration duration, country of origin), and prior research experiences (e.g., previous research experience, types of studies). Focus groups and interviews were audiotaped and conducted in private offices or a location of participants’ choosing (i.e., safe and confidential spaces where women felt they could share their perspectives)[46]. All participants received $10 USD in in-kind goods (e.g., telephone card or household/personal items of their choosing), condoms, HIV/STI prevention information, and referrals to medical care and social support services (e.g., HIV testing, hospitals, women’s shelters). All study materials and procedures (i.e., recruitment and informed consent procedures) were designed to assure and remind participants that our research team was not associated with government authorities such as police or immigration, as well as to assure participants of confidentiality of the data. For example, outreach workers explained to participants that confidentiality was protected through the use of study ID codes (rather than names), that all identifying information was removed from the transcripts by a team member prior to the analysis/dissemination, and that careful measures were in place to safeguard data storage.

Of the 33 study participants, 27 participated in a group session and 6 women who preferred to share their opinions privately participated in individual, face-to-face interviews. We conducted 10 focus groups, with between 2–5 women per group. Focus groups were led by experienced moderators/outreach workers who introduced topics and monitored group dynamics to facilitate representation of different perspectives [84], using group interactions to generate insights [85]. Focus groups and interviews were based upon a loosely structured guide that was iteratively revised to explore emergent themes, as previously reported[46].

Focus groups and interviews began with defining research.[46] Given that most prior research with sex workers in this community has been epidemiological (e.g., quantitative survey and HIV/STI serological testing), discussions focused on epidemiological, non-intervention research where possible. In brief, participants were asked to share examples of HIV research they were aware of or had previously participated in. Moderators were trained to clarify the differences between research and HIV/STI services (e.g., testing, educational workshops) to address challenges that emerged in operationalizing the concept of ‘research,’ particularly for participants without prior research experience. For example, during initial focus groups, when asked about experiences with research studies, some participants began to discuss their experiences with routine HIV/STI testing. These challenges were often linked to the fact that epidemiological studies are traditionally conducted at municipal clinics where HIV/STI care is provided. As previously described [46], this was addressed by operationalizing ‘research’ (vs. service provision) using examples of HIV research typically conducted with sex workers locally. Participants were asked to share examples of HIV research they were aware of (or previously participated in); facilitators clarified and explained the differences between research and HIV/STI services (e.g., testing, educational workshops) to address challenges in operationalizing the concept of “research.” As previously reported[46], during the interviews and focus groups, discussion topics and questions centred around the themes of barriers and facilitators of participation in HIV-related research (e.g., confidentiality, mistrust, anticipated benefits), related contextual influences (e.g., migration, work environments, manager/peer roles, interactions with police), and recommendations for future research (e.g., recruitment, researcher roles).

In addition to initial focus groups and interviews, 3 follow-up sessions (2 focus groups, 1 interview) were conducted with a subset of participants (*n* = 7). Women who expressed particularly diverse and/or strong opinions during initial focus groups or interviews were invited for follow-up sessions, which were used to more deeply explore and elaborate upon concepts that emerged during earlier interviews and focus groups, as well as to conduct “member-checking” (i.e, to gather participant feedback on preliminary findings and their interpretation).

### Data Analysis

Focus groups and interviews were transcribed, translated and accuracy checked by bilingual staff and personal identifiers were removed. Transcripts were managed and coded in NVivo Version 10 (QSR, Australia). Coding employed a detailed coding scheme that was collaboratively designed and iteratively revised by the principal investigator (SG) and project coordinator (TR). Using the constant comparative method,[86] we began with open coding to initially describe the structure and key themes that emerged in the data (e.g., key perceived benefits and risks related to participation in HIV research)[46]. As migration experiences emerged as a key issue that could promote or inhibit research engagement, resulting from the unique social and structural contexts experienced by migrant sex workers, we subsequently used axial coding to group and regroup the data until a subset of themes emerged which articulated key research-related ethical challenges and opportunities related to the central theme of migration. While nine participants were international migrants, an additional 21 were internal migrants; given that migration-related issues frequently emerged within discussions of research-related barriers and facilitators among migrants and non-migrants alike, our analysis included the full sample to fully elucidate the unique migration-related social and structural experiences and concerns that were articulated by sex workers during interviews and focus groups.

## Results

### Participant Characteristics

Participants’ median age was 29 years old and almost three quarters had primary school education or less (Table 1). Countries of origin included Guatemala (n = 24), other Central American countries (e.g., Honduras, El Salvador) (n = 7), and Mexico (n = 2) and among Guatemalan participants, the majority were internal migrants from primarily rural communities. Two-thirds of participants reported engaging in sex work in entertainment venues (e.g., bars, cantinas), and the remainder operated in a more mobile/independent capacity out of locations such as truck stops, parks, and hotels/motels. The majority (70.0%) were registered as sex workers. In terms of prior research participation, 20 women had previously participated in research; 19 had received HIV/STI testing, 14 participated in in-depth interviews, 10 participated in a quantitative survey, and only five had been involved in focus groups.

**Table 1:** Characteristics of participating female sex workers (*N* = 33), Tecún Umán, Guatemala, 2013–2014.

Variable	*n* (%)
Age, in years (median, range)	28 (20–48)
Education	
*None*	4 (12.1%)
*Some primary school/Completed primary school*	20 (60.6%)
*Some middle school/Completed middle school*	4 (12.1%)
*Some high school/Completed high school*	5 (15.2%)
Marital status	
*Single or Divorced*	27 (81.8%)
*Married*	4 (12.1%)
*Widowed*	2 (6.1%)
Migration status	
*Internal migrant*	21 (63.6%)
*International migrant*	9 (27.3%)
*Local resident (non-migrant)*	3 (9.1%)
Work environment	
*Entertainment establishment only (e*.*g*., *bar*, *cantina*, *or brothel)*	22 (66.7%)
*Independent/mobile (e*.*g*., *truck stop*, *park)*	11 (33.3%)
Registered sex worker	23 (70.0%)
Ever participated in an HIV research study	20 (61.0%)
Time since last research participation, in months (median, range)*	5.0 (0.1–12.0)
Instruments used in previous research participation*	
*Biological testing for HIV/STIs*	19 (95.0%)
*Survey or questionnaire*	10 (50.0%)
*In-depth interview*	14 (70.0%)
*Focus group*	5 (25.0%)

Note: All variables represent *n* (%) of participants unless otherwise stated.

*Among research-exposed women only (i.e., those who had previously participated in a research study) (*n* = 20)

### Thematic Results

Major themes emerging in this study included key ethical challenges and opportunities related to: mistrust and fear rooted in the social isolation frequently faced by recent migrants; intersecting concerns related to immigration status, criminalization, and sex work regulations; and migration-related benefits and risks of HIV/STI testing (e.g., immigration implications, stigma).

### “Almost Everyone Is Just Passing Through”: Mistrust and Fear Related to Transience, Mobility, and Social Isolation

International migrants typically left their hometowns and migrated Northbound in search of a better life for themselves and their families, fleeing poverty, violence, and gender inequalities. Most had migrated alone, and those with children had typically left them with family (e.g., grandmothers) in their home communities. For most, the period of initial arrival in Tecún Umán was characterized by intense social isolation, and most described feeling socially isolated as a powerful barrier to research participation (i.e., due to not knowing whether or not researchers are trustworthy).

Previous work with sex workers in this setting has elucidated the importance of rapport and the cultivation of meaningful relationships with research teams as a critical precondition for their ethical engagement in HIV research.[46] However, analysis of migrant sex workers’ narratives in the current study revealed how the social isolation experienced by recent arrivals to the community often resulted in mistrust and prevented opportunities for the development of rapport with researchers:

You come here as a newcomer, you don’t trust anyone and you can’t be talking about personal stuff. I think this can be a reason not to come here with you.

[International migrant, Honduras, 1 month since arrival]

High levels of mobility and the relatively short duration of stay of migrants in border communities such as Tecún Umán also limited opportunities for the establishment of meaningful relationships or rapport with other sex workers, service providers, and researchers. As the following participants noted when asked about the reasons that migrants might be reluctant to participate in research:

An immigrant is here one day and not the next. That is a problem because almost everyone is just passing through.

[Internal migrant, 22 years since arrival]

P2: Sometimes the girls are here not for long, a week, two weeks and… good-bye…they leave…they are just here for 8 days, 15 days, 20 days; the most they stay is a month, right?

P1: If they don’t like it, they only stay 2 to 3 days and good-bye.

Q2: So it would be difficult to establish contact with them if they’re here just a few days?

P2: Yeah.

[Internal migrants, 4 years and 8 days since arrival]

Some women also discussed how being a migrant offered some advantages in terms of the anonymity migrants experience in the community relative to local residents. Whereas local residents went to great lengths to conceal sex work involvement from family and community members, migrants without significant local social ties could be less concerned about the social consequences of disclosing sex work or other potentially stigmatizing behaviors as part of research participation:

I know a lot of people from other countries and they are even more open than Guatemalans…they would participate because since they are in Guatemala, no one knows them. Aha. Like they say, they don’t give a shit about it.

[Internal migrant, 22 years since arrival]

### “It’s the Fear You Feel Being a Foreigner”: Concerns Regarding Immigration Status

Participants in this study worked in a context characterized by frequent immigration and police raids of sex work establishments. Migrant sex workers’ concerns regarding HIV research participation often related to broader structural considerations including immigration status, migrant sex workers’ disproportionate vulnerability to punitive legal/policy consequences (e.g., arrest, deportation), and human rights abuses by government authorities. One participant explained: “They [migrants] do have more risk… because they don’t carry papers with them, do not have a way to identify themselves, they’re in the streets…Here in Guatemala, a foreigner is looked at [differently]”.

Fear of persecution by immigration authorities was a serious concern within migrants’ day-to-day lives, with profound implications for their ability to safely engage in research and HIV/STI services. All of the migrant sex workers interviewed were either undocumented or had transit permits, but lacked work authorization. As such, they greatly feared the potential impact of raids on their work establishments, which could lead to deportation (most had been previously deported). One participant noted:

If they [immigration] found out they’re Hondurans, they would take the poor girls away….here in Guatemala it is prohibited for foreigners to work.

[Internal migrant, 15 days since arrival]

Concerns regarding immigration status and fear of deportation were found to have direct and indirect consequences for the responsible conduct of research with migrant sex workers. Participants noted the tendency for international migrants to worry about the potential for researchers to be associated with immigration authorities–a sentiment most commonly expressed within the context of being approached by an unfamiliar research team. Women shared recent experiences in which this had been the case for their peers during recruitment for HIV-related studies:

The last time [her immigrant peers were approached by researchers], they felt distrust because they thought they were going to be turned in to immigration or something like that.

[Internal migrant, 3 months since arrival]

Findings also revealed that migrants often mistrusted researchers due to the possibility that sensitive information (e.g., regarding sex work, immigration status) might be shared with immigration authorities, resulting in negative immigration consequences. These considerations were often contextualized within discussions of the preference of migrants in another country to ‘stay under the radar’ (e.g., by limiting the development of social networks, avoiding discussions of immigration matters) to reduce the possibility of negative interactions with government authorities. For example, a recent arrival reflected on how such safeguards employed by international migrants could adversely impact their participation in research and other opportunities for social connections, further reinforcing social isolation and mistrust:

[You act] private, you don’t open up that much…because our fear is that they will send you back to your country with nothing.

[Undocumented migrant, Honduras, 5 days since arrival]

During fieldwork, our team observed firsthand the effects of such crackdowns and raids of sex work establishments in the region, noting the ways in which raids could amplify fear and mistrust among migrant sex workers. Interviews and focus groups conducted following crackdowns also indicated how these structural forces could enhance the ethical and practical challenges of conducting research with migrant sex workers:

They feel mistrust right now because immigration just came and they’re traumatized…This young girl who just arrived, I told her to come [to a focus group] but she was afraid the police might show up. I told her there was no danger…but she said no.

[Internal migrant, 4 years since arrival]

### “It’s Fear of the Police”: Intersecting Concerns Regarding Policing and Sex Work Regulations

Concerns regarding interacting with immigration intersected with migrant sex workers’ fear of persecution by police (e.g., related to their sex work involvement). Human rights abuses by police and immigration officials–including extortion, abuse of power, and physical/sexual violence–were described as an overarching concern during migration, as well as in the workplace upon arrival in migration destinations (i.e., Tecún Umán). While sex work remains criminalized in Guatemala, it has been historically tolerated under public health regulations that require workers in formal establishments (e.g., bars, clubs) to register and receive regular HIV/STI testing. While registered sex workers were often less susceptible to police abuse or harassment, those without registration greatly feared detention, abuse or extortion by police. An unregistered worker described the fear she experienced upon being approached by outreach workers to participate in research for the first time:

I felt bad…I asked, “Who are those people, and what if they are here to catch us?”…Because we don’t have any papers [proof of registration] to back us up, right, and the police may come and get us. I thought maybe one of you could say something to the police…yes, I was worried and afraid.

[Internal migrant, 7 years since arrival]

The threat of persecution by police emerged as a critical human rights theme that exacerbated ethical and practical challenges related to migrant sex workers’ research engagement. Though concerns such as policing have been previously shown to negatively impact both migrant and non-migrant sex workers,[21, 33, 36, 87] the unique structural circumstances faced by migrant workers (e.g., social isolation, lack of work authorization) exacerbated such concerns. For example, migrant sex workers’ often feared that researchers could be affiliated with the police, potentially resulting in negative consequences such as deportation:

One [peer] asked me [with respect to the researchers’ identities]…“but it isn’t the police, is it?” I told her it wasn’t at all! “How can you think it’s the police?” I asked. She said, “It’s just that I’m afraid that they might send me back to my country.” I said she shouldn’t be afraid, that she shouldn’t worry because it’s not the case…When I was first invited to come, I also thought badly. I thought it was the police too.

[International migrant, 7 months since arrival]

As a response to immigration-related fears and a desire to limit contact with government authorities, some migrants restricted their mobility, moving further outside the reach of researchers and health services:

They’re afraid to come. I told one of the newcomer girls not to be afraid, because when she saw the city police car she said: ‘there comes the police!’ She’s afraid to go out.

[Internal migrant, 4 years since arrival]

As previous researchers have noted, punitive policy and legal environments may ‘push’ difficult-to-reach populations (i.e., migrants, sex workers, people who use drugs, and trafficked persons) further underground, exacerbating challenges to research and health/social services delivery.[39, 67, 88, 89]

### Benefits and Risks of HIV/STI Testing for Migrant Sex Workers

We previously reported on how sex workers in Tecún Umán perceived the HIV/STI testing and prevention access (e.g., condom demonstrations and negotiation skills) typically offered by epidemiological HIV research studies to be potential benefits of research participation.[46] Here we focus on the ways in which migrant status was perceived to pose unique concerns and benefits for HIV/STI testing accessed within the context of research. HIV/STI testing and counseling was noted as particularly needed for recent arrivals from other countries and rural communities, who often had limited (if any) previous exposure to HIV/STI prevention or testing, as most had not engaged in sex work prior to their arrival and came from more conservative communities where access was constrained. When asked about the reasons that migrant sex workers might participate in research, the following participant noted access to HIV/STI testing and prevention to be critical motivators:

You come and you’re a newcomer and you get orientation about how to use a condom, it’s good to do HIV testing, to have all those tests done, because there are people who come here and have never worked in this business. So it’s good for newcomers to go.

[Internal migrant, 3 months since arrival]

However, HIV testing offered through research studies also carried potentially negative consequences due to the social, economic, and legal consequences of a potential positive test result[46]–concerns which were exacerbated for migrants. In addition to fear of loss of employment upon testing positive, migrants also expressed concern about deportation and the multiple forms of stigma that they anticipated a migrant sex worker living with HIV would be subject to:

There are some of us who say, “Ah, we are foreigners, and if we get the [HIV] tests done and they turn out bad, they’re going to send us to hell, because they’re going to say that we’re infecting people here and we’re not even from here. So, then they can go and tell immigration,”

[International migrant, Nicaragua, 2 years since arrival]

Our fear is that they will send you back to your country with nothing. You came over here with nothing, and returning with nothing—that’s our fear.

[Undocumented migrant, Honduras, 5 days since arrival]

Fear of negative consequences of HIV testing was often part of broader discussions of the ways in which sex work regulations have been historically enforced, including through raids and inspections of sex work venues. An international migrant described the ways in which police verified compliance with sex work regulations in indoor venues and how this intersected with immigration enforcement:

The police come and demand documents, tests, everything—they demand the HIV test and if they are from another country, they demand their passport…they wanted to arrest me, because I just arrived here…and I don’t have the papers.

[International migrant, Honduras, 4 years since arrival]

## Discussion

The current study elucidated the intersection between the human rights vulnerabilities of migrant sex workers and barriers to research participation (e.g., punitive immigration and sex work laws and policies, social isolation) to inform the responsible conduct of HIV research with migrant sex workers. Concerns related to migrants’ social isolation, legal immigration status, policing, and the enforcement of public health regulations surrounding sex work were found to pose powerful social and structural challenges to the ethical and meaningful engagement of migrant sex workers in research. While HIV/STI testing offered through research studies was acknowledged as highly beneficial (particularly for recent arrivals), this also raised concerns of negative consequences for migrants, including deportation and enhanced stigma. Participant narratives indicated that the combined effects of social isolation and fear of punitive interactions with government authorities (e.g., deportation, arrest) may not only limit migrant sex workers’ research engagement (especially in peer-based designs), but more broadly constrains their abilities to organize to advocate for improved health and working conditions and to access existing health and social services. Given that such punitive policy and legal environments have been previously shown to ‘push’ marginalized populations (i.e., migrants, sex workers, people who use drugs, and trafficked persons) further underground,[39, 67, 88, 89] peer-driven recruitment strategies may be particularly important for reaching migrant sex workers in future research. In addition to the focus of this analysis on the migration-specific barriers and challenges faced by sex workers, it is important to note that both migrant and non-migrant sex workers who participated in this study also discussed contextual factors that positively influenced their decision to participate in research[46]. For example, as previously described[46], a desire to improve one’s health and that of others, and the positive influence of rapport and trusting relationships established with outreach workers were frequently discussed as promoting research engagement, and these contextual influences should be taken into account alongside the findings of this analysis.

Our results support previous research highlighting the need for increased attention and sensitivity to the impact of local human rights conditions on research ethics.[90] Narratives regarding the negative impacts of policing and immigration raids on sex work venues are consistent with previous health research in this setting[76, 78, 91] and others (e.g., Northern Mexico, Canada),[8, 87, 92] while uniquely exploring the impact of such concerns on migrant sex workers’ research engagement. While some studies have described the importance of attending to cultural differences in HIV and substance-related research with migrants,[39] the influence of broader structural forces, including immigration status and criminalization, have been infrequently studied within migrant health research.

Our findings emphasize the critical importance of investigators’ knowledge and responsiveness to local human rights conditions when conducting research with marginalized populations in diverse global settings. Cultivating close community partnerships, consulting with a CAB that is knowledgeable about these issues, and ideally, developing and implementing research projects in close collaboration with migrant sex workers themselves are key ways investigators may be able to achieve this. Additional strategies that may assist in addressing perceived research risks include maintaining a continued and trusted presence in the community; incorporating strong reassurances and explanations of measures undertaken to protect confidentiality; and clearly and frequently emphasizing the investigators’ independence from police or immigration authorities. While important ‘best practice’ guidelines have been developed for HIV and STI service delivery with marginalized populations, including sex workers[93, 94], the development of ‘best practices’ for the responsible conduct of health research with migrant sex workers in Latin America and other low and middle-income contexts remains needed in light of their unique vulnerabilities and needs.

While previous research has articulated the social isolation often experienced by migrants and implications for migrant health (e.g., HIV-related risks, health access),[1, 8, 18, 95] our study documented the implications of migrants’ social isolation on researcher mistrust and research engagement. Although this remains an under-appreciated aspect of migrant health research, some investigators have previously written about the importance of establishing and maintaining rapport with migrant communities, noting the unique challenges posed by mobility (e.g., frequent changes in the migrant population and in residential locations). These challenges indicate the need for a more tailored and intensive approach to outreach in order establish and maintain rapport with migrants,[96] as well as the importance of meaningfully engaging and partnering with local organizations and health services that support migrant populations (e.g., shelters, public health clinics, NGOs) and maintain a more consistent presence within the community. This argument echoes earlier work by our team and others indicating the importance of rapport and the cultivation of reciprocal relationships between researchers and marginalized populations in global HIV research,[44–46, 54, 97] while illustrating the unique challenges to achieving this with migrant women.

Lastly, we identified both benefits and risks related to HIV/STI testing accessed through research studies for migrant sex workers. While testing offered free-of-charge was often perceived as highly beneficial, particularly for recent arrivals with limited access, participants also noted unique negative consequences of a positive HIV test for migrants to include increased stigma associated with perceptions of migrants as ‘vectors of disease’, deportation, or a loss of employment. Indeed, current sex work regulations in Guatemala and many other contexts in which sex work is regulated and HIV/STI testing remains mandatory prohibit sex workers from working upon an HIV-positive diagnosis or in the case of an untreated STI. Although Guatemala does not have any specific laws on the books that restrict the entry, stay, or residence of persons living with HIV, non-HIV specific laws and policies may be used in discriminatory and punitive ways to restrict the migration options of people living with HIV in Guatemala and neighboring countries (e.g., Mexico). For example, Mexico’s General Law for the Population reserves the right to deport foreigners who are considered a detriment to “economic or national interests” and lack the “mental or physical health” or “the necessary funds to support themselves.”

HIV/STI testing was problematized within broader discussions of the punitive enforcement of sex work regulations locally, exacerbating stress and fear related to HIV/STI testing for migrant sex workers. Non-coercive, voluntary HIV/STI testing services for sex workers that are supportive, non-stigmatizing, and respectful of sex workers’ autonomy and human rights remain critically needed.[98, 99] While policies surrounding this issue have recently changed in Guatemala (i.e., registration and police verification are no longer officially required), policies that continue to mandate compulsory HIV/STI testing of sex workers on the basis of public health concerns may continue to pose serious concerns for sex workers’ human rights and autonomy, particularly in the absence of appropriate safeguards to protect undocumented migrants and to ensure appropriate and supportive linkage to care.[100–102]

### Implications for Future Research and Interventions

Findings highlight the need for researchers to develop population-tailored and contextually sensitive procedures to address fears related to immigration and criminalization; reinforce positive and non-stigmatizing relationships with migrant sex workers; and support policy shifts that move away from punitive models towards approaches that are more congruent with promoting migrant sex workers’ rights and wellbeing. As in other settings, while significant efforts have been made by sex work organizations in Guatemala to promote recognition of sex work from a labour perspective, special efforts may be needed to engage migrant sex workers.

To promote ethical, valid, and relevant research with marginalized populations, and especially when conducting research in new or unfamiliar global contexts, collaboration and meaningful exchange between researchers and communities is needed.[103–106] Sex workers’ organizations have often used the phrase “*nothing about us without us”* to highlight the critical role of sex worker leadership and engagement to inform research, programs, and policies–a “best practice” that is embraced by HIV researchers in numerous settings.[25, 94, 106–111] However, this study identified the ways in which social isolation, fear, and mistrust among recent migrants can preclude the development of social trusts or research participation,[1] raising questions regarding how to best represent and engage migrant populations in health research. Past research with non-migrant populations has demonstrated the importance of peer-led or community-based models for enhancing the responsible conduct of community- and policy-relevant research.[44, 46, 103, 106, 110, 112, 113] As this issue has rarely been critically examined with respect to the engagement of highly mobile migrant populations in research, future efforts to identify appropriate models for the democratization of knowledge production and exchange among marginalized migrant populations remain needed (e.g., how to ensure meaningful community engagement in the design, conduct, and dissemination of research; feasibility of peer designs for mobile groups).

### Strengths and Limitations

Several potential limitations should be considered in interpreting our study results, including our sample composition. Firstly, there may be limitations to relying on accounts of those willing to participate in research to explore reasons for reluctance to research participation. Given that our findings were restricted to the perspectives and narratives of sex workers who agreed to participate in the current study, it is possible that the experiences of women with stronger concerns regarding research participation are under-represented. However, during interviews and focus groups, we asked questions pertaining to individual experiences in the past and present (i.e., reasons for reluctance to participate in the past), as well as broader peer and group experiences. In doing so, we were able to successfully elicit important insights regarding potential barriers to research for sex workers–including migrants (i.e., fear of police, mistrust)–who may have been underrepresented within our study sample. Additionally, while we engaged in both indoor and outdoor outreach to recruit a diverse sample of migrant and non-migrant sex workers to participate in the current study, a high proportion of participants within our sample identified as registered sex workers. While this may reflect the high proportion of venue-based SWs within the local context generally, since this study was completed, our team recently conducted an epidemiological study elucidating high unmet need for healthcare among unregistered, hard-to-reach sex workers who work in informal settings; future research investigating the experiences of this under-served group in terms of both research and service provision is recommended. Finally, we recognize that community and participant perceptions of our roles and expectations as researchers could result in an unwillingness to discuss problematic or unethical research practices; as part of our reflexive stance as qualitative researchers, moderators and research staff were trained to be aware of and mitigate this through numerous strategies, including cultivating an open and reciprocal interview atmosphere, ensuring that the PI was not present for all of the sessions, and engaging in long-term fieldwork to build trust within the community. Suggested approaches to mitigate this in future research could also include the application of long-term ethnographic methods, interviewing outreach workers and research staff about their experiences, and having individuals who have not previously engaged in research within the study community lead interviews or focus groups.

## Conclusion

In this study, social isolation, immigration status, policing, and the unique benefits and risks of HIV/STI testing for migrant women emerged as key challenges and opportunities related to the responsible conduct of HIV research with migrant sex workers. The development of procedures to address fears related to immigration and criminalization, and the cultivation of positive and non-stigmatizing relationships with migrant and sex work communities are recommended strategies to enhance the responsible conduct of HIV research with migrant sex workers, as are supporting community-led efforts to reduce stigma and foster community organization and supports for migrant sex workers.
